# How to Reduce the Latent Social Risk of Disease: The Determinants of Vaccination against Rabies in Taiwan

**DOI:** 10.3390/ijerph110605934

**Published:** 2014-06-04

**Authors:** Lee Ku-Yuan, Lan Li-Chi, Wang Jiun-Hao, Fang Chen-Ling, Shiao Kun-Sun

**Affiliations:** 1 Department of Bio-industry Communication and Development, College of Bio-resources and Agriculture, National Taiwan University, No. 1, Sec. 4, Roosevelt Rd., Daan Dist., Taipei 10617, Taiwan; E-Mails: howkaka@hotmail.com (L.K.-Y.); wangjh@ntu.edu.tw (W.J.-H.); kunsun@ntu.edu.tw (S.K.-S.); 2 Department of Business Administration, College of Business, National Taipei University, No. 151, University Rd., San Shia Dist., New Taipei City 23741, Taiwan; E-Mail: blueliki1214@hotmail.com; 3 Department of Banking and Cooperative Management, College of Business, National Taipei University, No. 151, University Rd., San Shia Dist., New Taipei City 23741,Taiwan

**Keywords:** social risk, rabies, vaccination, the theory of planned behavior

## Abstract

To control the latent social risk of disease, the government usually spreads accurate information and attempts to improve the public’s attitude toward adopting prevention. However, these methods with the Knowledge, Attitudes, and Practices (KAP) model do not always work. Therefore, we used the theory of planned behavior (TPB) to understand dog owners’ behavior and distinguished the knowledge effect as objective knowledge (OK) and subjective knowledge (SK). A total of 310 dog owners completed a questionnaire based on our model. We employed structural equation modeling to verify the structural relationships and found three main results. First, our model was fit, and each path was significant. People with better attitudes, stronger subjective norms, and more perceptive behavioral control have stronger behavioral intention. Second, perceived behavioral control, not attitude, was the best predictive index in this model. Finally, on perceived behavioral control, subjective knowledge showed more influence than objective knowledge. We successfully extended TPB to explain the behavioral intention of dog owners and presented more workable recommendations. To reduce the latent social risk of disease, the government should not only address dog owners’ attitudes, but also their subjective norms and perceptive behavioral control. Indeed, perceptive behavioral control and SK showed the most influence in this model. It is implied that the self-efficacy of dog owners is the most important factor in such a behavior. Therefore, the government should focus on enhancing dog owners’ self-efficacy first while devoted to prevention activities.

## 1. Introduction

Prevention is better than cure. Therefore, to reduce the social risk of disease, the government is devoted to many epidemic prevention activities, usually designed to increase the public’s rate of prevention behaviors. In these activities, the Knowledge, Attitudes, and Practices (KAP) model is the most commonly adopted. In general, the government spreads accurate information to increase the public’s willingness to adopt prevention behaviors. In some cases, these activities are effective, while in others, they are not, especially in unaffected areas, and this situation can cause a latent risk of disease outbreak. Therefore, we used rabies vaccination in Taiwan to exemplify and clarify this problem.

As one of many zoonoses that can be transmitted from other mammals to human beings, rabies causes high death rates worldwide. Rabies is a type of acute viral encephalitis, and because of its fast dissemination through the nervous system, few antibodies can affect immunoreactions. This leads to a death rate of almost 100%. If humans go untreated, they generally die of respiratory failure within 2–6 days [1]. Moreover, because of the long incubation period of rabies, a variety of hosts, and many paths of infection, proactive measures are necessary to prevent its occurrence and proliferation. Most reported cases of rabies occur in Africa, Asia, Latin America, and the Middle East. According to World Health Organization (WHO) estimates [2], rabies causes approximately 55,000 deaths annually, with Asia reporting 31,000 and Africa, 24,000.

Although, Taiwan currently does not have any human rabies cases, the occurrence rate of rabies in Asia is the highest in the world. Except for Japan, all the countries around Taiwan are epidemic areas, particularly China. In recent years, the number of rabies cases reported in China has rapidly increased, representing a severe threat than that of the Severe Acute Respiratory Syndrome (SARS). With cases increasing from 163 in 1996, to 3,215 in 2006, China reported more than 1,300 rabies deaths in 2012, a death rate among the highest of all infectious diseases [3]. With the Economic Cooperation Framework Agreement (ECFA) deal, more activities between China and Taiwan may lead to a greater likelihood of a rabies outbreak in Taiwan. An imported case from China, in July 2012, exposed this potential risk. If a rabies epidemic occurs in Taiwan, public health, social order, agriculture, and the tourism industry might suffer severely. For example, after the March 2003 SARS outbreak, the number of tourists visiting Taiwan decreased by 600,000 that year, leading to a loss equal to 13 billion US dollars in foreign exchange. Since a rabies epidemic could cause much more severe results than the SARS outbreak, implementing effective epidemic prevention policies is crucial.

For cases with a known infection source, canines are the most common culprits [2]. In other words, preventing canine rabies infection is important to the health of Taiwanese nationals as well as tourists. A 2005 report by WHO revealed certain conditions causing high risk of rabies infection: (1) a large number of infected canines; (2) ineffective canine management; (3) a canine vaccination rate below 80%, including stray dogs; and (4) people’s ignorance because of insufficient general knowledge and insufficient education budgets. Conversely, the effective investigation of epidemic situations, a high vaccination rate (over 80%), and effective canine management are key factors in successfully controlling rabies infections. According to the report, since 1973, WHO has widely promoted two rabies prevention measures: broad vaccination programs and strict control of stray dogs.

Among KAP-model promotions, improving knowledge and instilling positive attitudes toward prevention should be the main elements of a Taiwanese rabies-prevention campaign. However, Taiwan’s current vaccination rate is only between 30% and 40% [4,5], far below WHO’s 80% recommendation [2]. To close this gap, we focused on owners’ intentions to vaccinate dogs and attempted to find the determinants for vaccinating against rabies in Taiwan. We had three main purposes: first, we argued that the KAP model’s attitude concept should be extended to behavioral intention and that the theory of planned behavior (TPB) would be a suitable structural model for behavioral intention of vaccination against rabies because of its wide application in research on forecasting and building rational behavior. TPB has been used to predict, for instance, people’s intention to vaccinate against influenza as well as many other health behaviors [6]. We used structural equation modeling (SEM) to verify that TPB can explain people’s intention to vaccinate dogs against rabies [7]. Second, we argued that attitude is not the only key factor, or even the best factor, for understanding dog owners’ behavioral intention. According to the low vaccination rate in Taiwan, we considered only improving knowledge and instilling a positive attitude are not enough to control the latent risk of rabies in Taiwan. By applying TPB to the intention of rabies vaccination, we described relationships among the variables to determine the best predictive index. Finally, we argued that the knowledge concept of the KAP model should be distinguished into two types, objective knowledge (OK) and subjective knowledge (SK). OK indicates the level of accurate information in one’s cognition about the target; SK indicates the level of one’s perceptions of what or how much one knows about the target [8,9]. Azjen *et al.* [10] argued that knowledge, especially objective knowledge, would affect attitude and enhance self-efficacy (perceived behavioral control, PBC), but they did not test the correlation between subjective knowledge and TPB. Besides, we thought that especially SK, rather than OK, would be more likely to prompt people to vaccinate their dogs. In conclusion, we proposed to the government rabies prevention policies and suggestions for raising the vaccination rate through TPB, thus not only helping prevent a rabies outbreak in Taiwan but also preventing latent risk for similar situations.

## 2. Literature Review

### 2.1. Behavior for Vaccination against Rabies

Vaccination is a health behavior that consists of a personal act to preserve or strengthen one’s health [11,12,13]. Many methods have been employed to increase vaccination rates, for example, through increased knowledge and better attitudes, but these strategies have shown only limited success [14,15]. Descriptions of vaccination determinants have been mainly from physician perspectives, and past studies have often ignored those who actually make the decisions [15]. Hence, the limited success of these interventions clearly indicates the need for a fresh approach and new methods.

Besides, vaccinating one’s dog is not purely a health behavior. It includes a variety of factors: health, emotions, risk aversion, social perception, and so on. From the human perspective, dogs may be movable property, personal goods, and beloved pets, and those who vaccinate dogs against rabies may do so for one or more of the following motivations: enhancing their dogs’ health; loving their dogs; perceiving the risk of rabies; thinking other people hope they will; and others. Furthermore, vaccinating dogs is not only a personal act but also a social behavior involving moral perception. At least partly because authorities, such as WHO and the Center for Disease Control (CDC), have advocated epidemic prevention for several years; for some people, vaccinating dogs has become an ethical and moral act of socialization.

### 2.2. Theories for Understanding Behavior of Vaccination against Rabies

Applications of certain theoretical frameworks have manifested as well suited to the design of health behavioral change interventions [6,14,15]. Among theories commonly used to understand health behavior [14,15,16] are the theory of reasoned action (TRA) and the TPB that have effectively explained inventions and induced health behavior changes [14,15,16]. These two theories have increased understanding of the processes involved in vaccination decision-making at the individual level. Constructed by Ajzen (1991) [17], the TPB is an especially well established framework for predicting various types of health behaviors [6].

A central element in TRA and TPB is the individual’s intention to perform a given behavior [17]. Previous research shows the instant antecedent of any behavior to be the intention to perform that behavior. People who have a stronger intention to act are more likely to perform the behavior [18,19,20], especially reasoned actions and planned behaviors.

Developed by Fishbein and Azjen, the TRA is based on two assumptions. The first is that intentions best predict behaviors, and the second is that human behavior is quite rational and employs the limited information available to the individual [21,22]. In this theory, two independent factors determine one’s intention: attitudes and subjective norms. Attitudes consist of general evaluations of behavioral performance and beliefs about the consequences of performing the behavior, weighted by an individual’s evaluation of each consequence. Subjective norms reflect general perceptions of social pressure to perform the target behavior and are affected by the expectations of important referents, weighted by an individual’s motivation to comply with each referent. Researchers have conducted many empirical studies on this topic over the past 30 years and provided evidence in support of TRA to explain health and social behavior [23,24,25,26,27,28].

However, although previous studies have successfully verified that TRA is helpful in predicting intention and behavior, other studies have revealed its limitations [17,29,30]. For the most part, the behaviors investigated through TRA have been subject to considerable volitional control [31]. Some studies using the health belief model, the source of all health behavior change models [32], have added self-efficacy to their models [31,32,33].

In contrast to TRA, TPB contains perceived behavioral control, including the concept of self-efficacy [17,29]. Due to TRA’s limitations, Ajzen developed an enhanced behavior prediction model in which the individual may not have considerable volitional control or be able to perform well [17,29]. Ajzen believed that the construct of perceived behavioral control is belief-based, similar to attitudes and subjective norms in TRA [34,35,36]. Perceived behavioral control represents one’s belief about how easy or difficult it is to perform a behavior and is easily measured with a questionnaire [29,30].

Using the TRA as a base, Ajzen constructed TPB to incorporate perceptions of control over performance of behavior as an additional predictor [17,29]. He then used TPB to predict behavior that an individual may not be able to perform at will [20]. Ajzen also proposed that perceived behavioral control affects behavior not only indirectly through intention but also directly [17,29]. In fact, many theoretical and empirical studies provide evidence supporting TPB. In 1985, Ajzen presented “*From intentions to actions: A theory of planned behavior*” to open theoretical discussions about TPB. He theorized that the relationship between behavioral intention and behavior is stronger when perceived behavioral control is high. To provide a powerful foundation for TPB, he also presented arguments about social psychology [34], organizational behavior [17], self-efficacy [36], laws of human behavior [37], and the relationship between consumer attitudes and behavior [38]. In addition to this psychological research, Armitage and Conner argued that TPB can be applied to health behavior and also disseminated TPB to such fields as moral behavior, technological behavior, and exercise behavior [39]. These researchers found that TPB explained an average of 39% of the variance in intention and 27% of the variance in behavior.

The TPB concept has received strong empirical support in applications to a variety of domains. Nevertheless, the current study is one of only a few attempts to use TPB as a conceptual framework for vaccination, and more specifically, canine vaccination against rabies. This behavior involves morals, social impressions, and health concepts. Researchers have repeatedly used TPB to interpret moral behavior [40], including behaviors of health promotion [41], environmental friendliness [42], and tax compliance [43]. In the social behavior domain, researchers used TPB to examine alcohol abuse [44], volunteer behavior, substance use [45], blood donations [18], and others. In the health domain, TPB has explained various behaviors, for example, smoking [46,47,48], giving up smoking [49], and drinking [48,50]. Researchers have also used TPB to predict a variety of attendance decisions for many types of health behaviors, including the decision to attend health checks and health clinics [51,52], breast cancer screenings [53], and workplace health and safety courses [54]. Therefore, TPB could be the most powerful theory for predicting a rise in the rate of vaccination against rabies.

However, despite the fact that TPB has never been applied to explain the behavior of vaccination against rabies, it has been applied to predict of a wide range of other behaviors in previous research, including health behavior, social behavior, and moral behavior, and these behaviors resemble the targeted behavior’s concepts. Therefore, we employed TPB to construct a theoretical framework for explaining the behavior of owners’ ensuring that dogs are vaccinated against rabies.

As in the original TRA, intention determines actual behavior [17,29]. Intentions are assumed to measure motivational factors that influence a behavior; they are indications of how hard people are willing to try and how much effort they are planning to exert to perform the behavior [17,29]. Furthermore, intention is jointly determined by attitudes, subjective norms, and perceived behavioral control in the TPB model. First, attitudes refer to the degree to which an individual favorably or unfavorably evaluates the behavior in question; second, subjective norms refer to social pressure to perform or not to perform the behavior; and, third, perceived behavioral control refers to whether the individual anticipates the action’s performance as relatively easy or difficult. Presumably, this third measure reflects past experiences and anticipated hindrances. Generally speaking, a person with a more favorable attitude, more positive subjective norms, and higher perceived behavioral control has a stronger intention to perform the target behavior [17,29].

Based on TPB, we argue that behavioral intention is determined by an individual’s attitudes toward rabies vaccination, subjective norms about this behavior, and perceived behavioral control, *i.e.*, whether one can control taking a dog to receive the rabies vaccine. In other words, this study hypothesizes that favorable attitudes, high subjective norms, and good perceived behavioral control enhance the behavioral intention of rabies vaccination. Beside, Ajzen argued that individual behaviors sometimes could be predicted best by self-efficacy, especially while the behaviors need to be controlled [55,56]. In taking a dog to receive the rabies vaccine, we also considered perceived behavioral control, not attitude, would be the best predictive index:

H1: Attitude (A) toward the vaccination of rabies positively affects behavioral intention (BI).
H2: Subjective norms (SN) about the vaccination of rabies positively affect BI.
H3: Perceived behavioral control (PBC) over vaccination positively affects BI.
H3b: the PBC effect is greater than the attitude effect.

### 2.3. The Knowledge Effect on Attitude and Perceived Behavioral Control

Knowledge changes people’s cognition and affects their behavior [57]. Knowledge can be defined as a kind of stored information, which people obtain and acquire from processing data [58]. However, in previous studies, knowledge has been discussed according to two concepts, objective knowledge (OK) and subjective knowledge (SK) [8,9].

OK indicates the level of accurate information in one’s cognition about the target; SK indicates the level of one’s perceptions of what or how much one knows about the target [8,9]. The two concepts are related, but must be distinguished: Specifically, people cannot actually recognize whether their perceptions of how much they know are correct. In other words, a cognitive gap usually exists between OK and SK. Moreover, OK can be measured by objective scales, but SK relates more to one’s self-confidence [8,59]. In this study, we defined the level of dog owners’ accurate information about rabies as OK and the level of dog owners’ perceptions of how much they know about rabies as SK.

People use their knowledge to develop a cognitive system and to judge whether to perform a specific behavior [10]. In the KAP model, people improve their preventive attitudes as they raise their knowledge of disease. When people receive accurate information about prevention of diseases, they know what coping behaviors should be taken and improve their attitudes about performing these behaviors [60]. In this study, we also presumed that owners will have better attitudes about taking their dogs to be vaccinated when they possess greater objective knowledge. People who have higher OK will have more positive attitudes about taking their dogs to be vaccinated.

H4: Objective knowledge (OK) about rabies positively affects attitude about rabies prevention.

**Figure 1:**
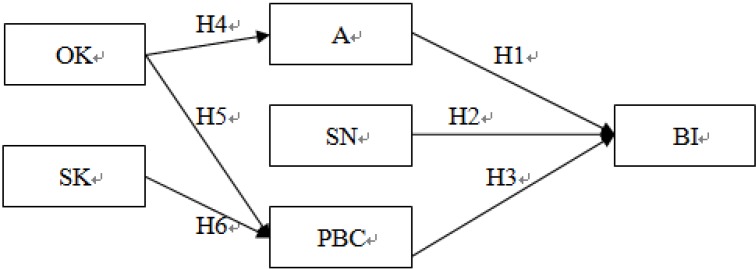
The hypotheses in this study.

Perceived behavioral control, including the concept of self-efficacy, is the distinguishing feature of TPB. When people do not have considerable volitional control or are not able to perform well, perceived behavioral control becomes a good predictor for explaining behavioral intention [17,29]. Ajzen argued that perceived behavioral control represents one’s belief about how easy or difficult it is to perform a behavior and that the construct of perceived behavioral control is belief-based [34,35,36]. An individual with knowledge about specific behavior reduces feelings about impediments and increases perceived behavioral control [17,29]. In other words, when people have enough knowledge about rabies and about vaccinating their dogs, they add self-efficacy and then feel good about performing the behavior. Specifically, both OK and SK can affect the ability to perform a specific behavior but with different mechanisms [9]. OK provides information and skill, reducing the impediment of bringing dogs to be vaccinated; SK enhances dog owners’ self-efficacy so that they feel they can perform the behavior well. Therefore, we argued that people with higher levels of OK and SK will have higher perceived behavioral control. Furthermore, Azjen *et al.* [10] argued that knowledge would positively affect attitude and perceived behavioral control. However, they did not test the correlation between SK and TPB. Because SK combines knowledge and self-confidence, it is more important in problem-solving [61,62]. Hence, we also argued that SK could affect dog owners’ perceived behavioral control more than OK. Figure 1 displays the proposed hypotheses for this study:

H5: Objective knowledge (OK) about rabies positively affects perceived behavioral control (PBC) about rabies prevention.
H6a: Subjective knowledge (SK) about rabies positively affects PBC about rabies prevention.
H6b: the SK effect is greater than the OK effect.

## 3. Materials and Methods

### 3.1. Questionnaire

This study administered a questionnaire to assess: (1) attitude; (2) subjective norms; (3) perceived behavioral control; (4) behavioral intention; (5) objective knowledge; (6) subjective knowledge; and (7) basic demographic data. The first four scales were revised from the sample TPB questionnaire designed by Icek Ajzen. Four sections evaluated attitudes, subjective norms, perceived behavioral control, and behavioral intention as to whether owners would take their dogs to receive the rabies vaccine injection. The last two scales (OK and SK) rested on the literature of objective knowledge and subjective knowledge and were revised by the outcome of export pretesting.

*Intention.* This study used three items with a 5-point semantic differential scale to measure participants’ intentions to have their dogs vaccinated. First, the statement, “I would like to take my dog to have the rabies vaccine injection” was rated on a 5-point semantic differential scale ranging from extraordinarily impossible (1) to extraordinarily possible (5). Second, “I will take my dog to have the rabies vaccine injection in the near future (3 months)” was rated on a 5-point semantic differential scale ranging from absolutely incorrect (1) to absolutely correct (5). Lastly, “I plan to take my dog to have the rabies vaccine injection in the near future (1 year)” was rated on a 5-point scale ranging from absolutely incorrect (1) to absolutely correct (5).

*Attitudes.* This study used three items with 5-point scales to assess attitudes toward the behavior. The scales ranged from strongly disagree (1) to strongly agree (5). These items were modified from “*Constructing a TPB Questionnaire*” by Ajzen and included “For me to take my dog to have the rabies vaccine injection is good,” “For me to take my dog to have the rabies vaccine injection is beneficial,” and “For me to take my dog to have the rabies vaccine injection is helpful.”

*Subjective norms.* To assess subjective norms, this study used three 5-point scale items ranging from strongly disagree (1) to strongly agree (5). We not only focused on the opinions of participants’ relatives and friends about taking their dogs for the rabies vaccine injection, but considered whether they would like to do so. These items included: “My family thinks I should take my dog to have the rabies vaccine injection,” “My friends think I should take my dog to have the rabies vaccine injection,” and “My relatives and friends have taken their dogs to have the rabies vaccine injection.”

*Perceived behavioral control.* This study used three items with 5-point scales to measure perceived behavioral control. These items were also adapted from Ajzen’s “*Constructing a TPB Questionnaire*.” The item “To bring my dog to have the rabies vaccine injection every year” was rated on a 5-point semantic differential scale ranging from “I can’t make this happen” (1) to “I can make this happen” (5); the item “I have the ability to take my dog to have the rabies vaccine injection” was rated on a 5-point semantic differential scale ranging from completely incorrect (1) to completely correct (5); and the item “To bring my dog to have the rabies vaccine injection” was rated on a 5-point semantic differential scale ranging from “I have no control over this” (1) to “I have control over this” (5).

*Objective knowledge.* Aligning with the CDC and WHO reports [1,2], we designed 17 items with key information on rabies. After our pretesting, we performed item analysis and deleted 7 items. Finally, 10 items were used to evaluate rabies knowledge in the “the objective knowledge of rabies index.” Each item employed a dichotomous scale (Yes or No question). We summarized ten scores to represent the objective knowledge of dog owners.

*Subjective knowledge.* According to the literature, subjective knowledge can be measured as a kind of self-confidence [8,59]. We took one subjective knowledge item from a self-report and three subjective knowledge items to assess the respondent’s self-confidence of rabies knowledge as compared with other dog owners, pet traders, and prevention experts. We used a Likert 5-point scale to measure the score, ranging from strongly disagree (1) to strongly agree (5).

### 3.2. Pre-Testing and Sampling

This study’s questionnaire in this study was reviewed by 10 epidemic prevention experts and staff members selected from among veterinary professors in universities and personnel at the bureau of animal and plant health inspection and quarantine. Besides that, it was pretested on 133 dog owners in Taiwan. According to their suggestions, we revised some items.

The geographic scope of this study is Taiwan and the Kimen district. We distributed the samples around Taipei, Taichung, Kaohsiung, Taitung, and Kinmen. To increase the response rate, each participant received a questionnaire accompanied by a gift valued at one US dollar. In total, 310 participants completed the questionnaire. The respondents were almost equally male (163; 52.6%) and female (147; 47.4%). Their age ranged from 16 to 73, with an average age of 37.6 years old (with a standard deviation of 12.33 years). As for the level of education completed, 12.9% (*N* = 42) had a junior/senior school degree, 72.6% (*N* = 225) had a bachelor’s degree, 11.9% (*N* = 37) had a master’s degree, and 1.9% (*N* = 6) had a doctorate degree.

## 4. Results

For this study, we used SEM to verify whether TPB can explain the intention of people to have their dogs vaccinated and whether knowledge of rabies can positively affect people’s attitude and perceived behavioral control. Besides that, we tried to review the relationships of these variables and find a determinant to explain the dog owners’ intention. We employed LISREL 8.7 to achieve this goal.

### 4.1. The Measurement Model

According to the hypotheses, based on TPB and KAP, there are six latent variables in this study: objective knowledge (OK), subjective knowledge (SK), attitudes (A), subjective norms (SN), perceived behavioral control (PBC), and behavioral intention (BI). Table 1 shows the means and standard deviations of all variables, for which there were no significant differences in gender, age, and education level.

**Table 1:** The means and standard deviations of the latent variables.

	A	SN	PBC	BI	OK	SK
M	4.44	4.14	4.48	4.25	6.6	3.20
SD	0.72	0.79	0.82	0.93	1.23	1.21

To evaluate internal consistency, we used Cronbach’s α to test the reliability of A, SN, PBC, BI, and SK to obtain rabies vaccines. In this study, the Cronbach’s α for A was 0.903, for SN was 0.839, and for PBC was 0.940. For the BI scale, it was 0.884, and for the SK scale, 0.945. All values of Cronbach’s α exceeded 0.80, and are thus well within the commonly accepted range of reliability [7,63] (Table 2).

Convergent validity can be determined by reviewing the average variance extracted (AVE) and composite reliability (CR) for each construct. This value should exceed 0.5 for average variance extracted and 0.7 for composite reliability [7,63]. In this study, all values of AVE and CR were greater than 0.639 and 0.841, respectively, well within the acceptable range (Table 2), thus providing evidence that the convergent validity in this study is acceptable.

**Table 2:** Reliability and convergent validity.

	Cronbach’s α	AVE	CR
A	0.903	0.764	0.906
SN	0.839	0.639	0.841
PBC	0.940	0.843	0.942
BI	0.884	0.678	0.862
SK	0.945	0.802	0.942

The AVE can also measure discriminant validity. Discriminant validity is acceptable when the AVE score is greater than the squared correlation coefficients among variables. In this study, AVE scores, showing in diagonal in Table 3, were all greater than squared correlation coefficients, confirming discriminant validity.

**Table 3:** Discriminant validity.

	A	SN	PBC	BI	SK
A	**0.764**				
SN	0.462	**0.639**			
PBC	0.397	0.303	**0.843**		
BI	0.410	0.372	0.410	**0.678**	
SK	0.107	0.168	0.124	0.104	**0.802**

### 4.2. The Structural Model

This study used SEM to examine the structural relationship of our model based on TPB and KAP and to determine the factors that are keys for owners’ intention to take their dogs for rabies vaccine. Besides, Ajzen argued that there could be some correlation among attitude, social norm and perceived behavioral control [17,29]. Several literatures also found evidence to support the relations among these variables [64,65]. Hence, according to the Modification Indices (MI), we opened the correlation among these variables in TPB. Finally, all indicators used to test the fitness of SEM models were acceptable (Table 4). It is shown that the fitness of our model was confirmed.

**Figure 2:**
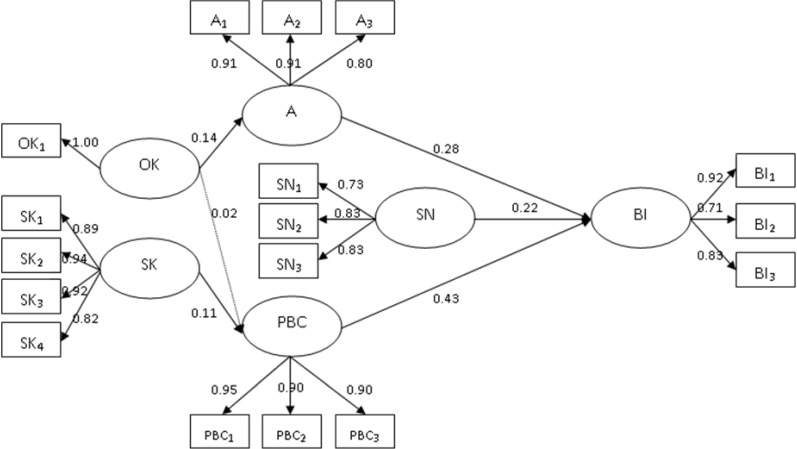
The SEM result.

Figure 2 illustrates the SEM results of our model. Almost all the paths and relations were significant, and the hypotheses were supported, except H5. For the A–I path (H1), the standardized coefficient was 0.28, with a *t*-value of 2.60 (*p* < 0.01). Hence, the stronger the attitudes, the stronger the behavioral intention to take a dog to be vaccinated against rabies. For the SN–I path (H2), the standardized coefficient was 0.22, with a *t*-value of 2.47 (*p* < 0.05). Accordingly, people who felt more social pressure and had higher subjective norms exhibited stronger intention to take their dogs to be vaccinated. For the PBC–I path (H3), the standardized coefficient was 0.43 with a *t*-value of 6.22 (*p* < 0.001). In other words, subjects with a higher sense of behavioral control had a higher intention to take their dogs to be vaccinated against rabies. Therefore, people who had a more positive attitude, stronger subjective norms, and more perceptive behavioral control would have stronger behavioral intention to take their dogs for vaccination against rabies. Additionally, three indices explained 69% of the variance on behavioral intention. Social norm positively affected attitude and attitude positively affected perceived behavioral control individually.

**Table 4:** The fitness of the model.

Index	Criteria	Result
χ^2^/df	<5	1.64
GFI	>0.9	0.94
AGFI	>0.8	0.91
NFI	>0.9	0.98
NNFI	>0.9	0.99
CFI	>0.9	0.99
IFI	>0.9	0.99
RFI	>0.9	0.98
PNFI	>0.5	0.77
PGFI	>0.5	0.65
RMSEA	<0.08	0.046
RMR	<0.05	0.042
SRMR	<0.05	0.032

In knowledge effect on attitude and perceived behavioral control, we also obtained evidence to support our hypotheses. For the OK–A path (H4), the standardized coefficient was 0.14 with a *t*-value of 3.11 (*p* < 0.05). In other words, the dog owners with higher objective knowledge had a more positive attitude toward taking their dogs to be vaccinated. For the OK–PBC path (H5), the standardized coefficient was 0.02 with a *t*-value of 0.50 (*p* > 0.05); for the SK–PBC path (H6), the standardized coefficient was 0.11 with a *t*-value of 2.29 (*p* < 0.05). In other words, people who have more subjective knowledge enhance their perceived behavioral control to perform rabies prevention. Therefore, for vaccinating a dog, objective knowledge enhanced an individual’s attitude and subjective knowledge enhanced an individual’s perceived behavioral control individually.

Furthermore, according to the path analysis result, perceptive behavioral control was the most obvious predictor of behavioral intention for vaccination against rabies (H3b); subjective knowledge effect on perceived behavioral control was greater than objective knowledge effect (H6b). That is to say that attitude is not the best factor and subjective knowledge must be considerable, for understanding dog owners’ behavioral intention. Therefore, when we are devoted to raising the vaccination rate against rabies, we need to revise the traditional KAP model, which only contains attitude and objective knowledge.

## 5. Conclusions

Vaccinating dogs is the most effective way to prevent an outbreak of rabies. Recently, there have been some animal cases but no human cases, in Taiwan. However, many latent risks still surround this area, especially those coming from China. With the ECFA deal, more activities between Taiwan and China could lead to a higher chance of a rabies outbreak in Taiwan. Although the administration has tried to improve knowledge and instill positive attitudes, the vaccination rate in Taiwan is still between 30% and 40%, far below the 80% rate recommended by the WHO. Hence, it is necessary to better understand and predict owners’ behavior about vaccinating their dogs.

In this study, we tried to integrate KAP and TPB to achieve the main goals. SEM results showed all the indices are acceptable and confirmed the fitness of our model. This means that our model is suitable not only for measurement but also for exploring the behavioral intention of vaccination against rabies.

To explain behavioral intention through TPB, each path was significant; this supported Hypotheses 1–3. In other words, people with more positive attitudes, stronger subjective norms, and more perceived behavioral control have stronger behavioral intention to vaccinate their dogs. Through these results, we verified that TPB is a suitable structural model for the behavioral intention of vaccination against rabies and successfully extended TPB to explain the behavioral intention of dog owners. In addition, perceived behavioral control, not attitude, is the most obvious index for predicting the target behavioral intention. In other words, the results confirm our argument that attitude, although important, is not the best index for understanding dog owners’ behavioral intention. Whether the Taiwanese vaccinate their dogs is mostly related to their belief about how easy or difficult it will be to accomplish.

To understand the knowledge effect on preventive behavior, SEM results also supported Hypothesis 4 and 6. People who had more objective knowledge of rabies tended to have more positive attitudes about taking their dogs to be vaccinated. As in the KAP model, objective knowledge could strengthen attitudes about prevention and provide the accurate information and skill that reduces impediments to vaccination. At the same time, people who had more subjective knowledge tended to have a better perceived behavioral control toward vaccination. In other words, subjective knowledge could change people’s perceptions and considerations about preventing rabies and enhance their self-efficacy so that they feel they perform the behavior well. Furthermore, results confirmed our argument that subjective knowledge showed greater influence than objective knowledge on perceived behavioral control: If we want to improve the vaccination rate by raising the dog owners’ perceived behavioral control, enhancing their subjective knowledge is more effective than providing greater objective knowledge.

## 6. Discussion and Suggestions

According to these findings, we made some contributions in both theory and practice. First, we successfully extended TPB to explain behavioral intention not planned only concerning the individual. This study is the first one to use TPB as a conceptual framework for canine vaccination against rabies. Previous authors successfully applied TPB in studies regarding the intention of people to obtain vaccinations for themselves. This study proves that TPB provides adaptability, in that people decide to perform some behavior for their dogs. Besides, for the KAP model, we also found the evidence to support it can describe the behavior against rabies. However, we found other important factors should be considered at the same time. Therefore, we should extend the KAP model to the Knowledge, Intention, Practices (KIP) model and take care, at least, of perceived behavioral control, attitude, and subjective norms.

Second, we found that when people decide to perform this kind of behavior, perceived behavioral control might be the most important factor. This result suggests that when people must make a decision outside of their own control, they might not feel that they can have considerable volitional control. Their control was a primary determinant of their behaviors [55,56]. In other word, for the TPB model, we also found evidence to support the argument of Ajzen. Furthermore, for the KAP model, we thought when the owners of dogs, or other animals, try to bring them to be vaccinated against diseases, the perceived behavioral control of owners should be more important than the attitude of them.

Finally, we found that subjective knowledge more greatly influences perceived behavioral control, the most obvious predictor of behavioral intention for vaccination against rabies, than objective knowledge. Ajzen *et al.* argued that knowledge is positively correlation with attitude and perceived behavioral control [10]. We not only found the evidence to support their argument but also pointed out the mechanism more clearly. We added the concept of subjective knowledge in our model and found the SK–PBC–BI path should be the most effective method for raising the vaccination rate against rabies. In other words, SK–PBC–BI-Practices path would be better than the traditional KAP model. Self-efficacy plays the key factor in such preventive behavior. People with greater self-efficacy feel more subjective knowledge and perceived behavioral control, and then they perform better.

Therefore, in order to reduce the latent social risk, the government should first focus on raising perceived behavioral control toward behavior. According to our findings, perceived behavioral control was the primary factor influencing behavioral intention. In other words, an effective epidemic prevention policy must be aimed at this factor. To influence perceived behavioral control, the government should provide manageable conditions and a comfortable situation, for instance, a vaccination subsidy, a more convenient location for vaccination, and so on. Moreover, social norms and attitude should also be addressed. Owners who consider vaccination necessary within a social atmosphere and believe that vaccination against rabies is beneficial will have stronger intentions toward this behavior. At the same time, subjective knowledge plays an important role on positively affecting perceived behavioral control. In other words, when we are devoted to epidemic prevention activities, enhancing dog owners’ self-efficacy is more important than confirming their learning. We should first address people’s confidence about rabies knowledge, and then confirm how much knowledge they actually have.

Furthermore, our results not only offer the government a reference for disaster prevention but also present some interesting directions for further research. The TPB model obtained a 69% prediction rate for dog owners’ behavioral intention, which still leaves 31% unexplained. In other words, people who tend to vaccinate their dogs are influenced by other factors, including risk perception, good care of dogs, and temporal immediacy. These may affect not only the intention to vaccinate but also the behavior’s practical execution.
